
JOURNAL INFORMATION
==============================
NLM Title Abbreviation: ScientificWorldJournal
Journal ID: ScientificWorldJournal
NLM Journal ID: 101131163
Journal ID: TSWJ

The Scientific World Journal

ISSN: 2356-6140
EISSN: 1537-744X
Publisher: Wiley

ARTICLE INFORMATION
==============================
PMCID: PMC6084030
PMID: 30147532
DOI: 10.1100/tsw.2001.190
Article ID: 253579
Article version: 1
Subjects: Short Report

Sequential Events During Nuclear Apoptosis

Kihlmark Madeleine
Imreh Gabriela
Hallberg Einar *
Södertörns Högskola (University College), Box 4101, 141 04 Huddinge, Sweden
*Einar Hallberg: einar.hallberg@sh.se
Publication date: 2001
Electronic publication date: 2001 Oct 23
Volume: 1
First page: 61
Last page: 61
Copyright: Copyright © 2001 Madeleine Kihlmark et al.
Copyright year: 2001
License: This is an open access article distributed under the Creative Commons Attribution License, which permits unrestricted use, distribution, and reproduction in any medium, provided the original work is properly cited.
License URL: https://creativecommons.org/licenses/by/3.0/

Funding: Swedish National Board for Laboratory Animals

==============================
Miami *Nature Biotechnology* Short Reports
*TheScientificWorld* (2001) 1 (S3), 61SR
ISSN 1532-2246; DOI 10.1100/tsw.2001.190

### SEQUENTIAL EVENTS DURING NUCLEAR APOPTOSIS

Madeleine Kihlmark, Gabriela Imreh, and Einar Hallberg*

Södertörns Högskola (University College), Box 4101, 141 04 Huddinge, Sweden
* Einar.Hallberg@sh.se

**INTRODUCTION.** Nuclear apoptosis is characterized by condensation and fragmentation of chromatin and disruption of the nuclear pores, membranes and lamina (1,2). We have investigated the sequential degradation of proteins from the nuclear envelope and correlated it to other hallmarks of apoptosis such as chromatin condensation, DNA fragmentation and nuclear pore clustering.

**METHOD.** Monolayer BRL cell cultures were treated with 0.5 µM staurosporine to induce programmed cell death. Degradation of nuclear envelope proteins was studied by immunofluorescence microscopy, confocal microscopy and Western blotting. DNA fragmentation was monitored using the TUNEL assay.

**RESULTS.** By Western blotting and pairwise comparison of double labeling immunofluorescence microscopy of BRL cells undergoing apoptosis we demonstrate that during apoptosis proteins of the nuclear envelope disappear in a specific order. POM121 (an integral pore membrane protein) and RanBP2 (a component of the cytoplasmic filaments of the NPC) disappeared first, followed by NUP153 (a component of the nucleoplasmic basket) and lamin B, whereas p62 (centrally located in the nuclear pore) remained throughout the experiments. The apoptotic degradation of proteins from the nuclear envelope could be totally prevented by a caspase inhibitor.

**DISCUSSION.** Our data suggests a specific order of events during nuclear apoptosis, summarized in Fig. 1. Condensation of chromatin occurs before DNA fragmentation, degradation of nuclear envelope proteins and disruption of the nuclear membranes. Degradation of POM121 and RanBP2 started before DNA fragmentation could be detected. At a later step NUP153 and lamin B was degraded concomitant with onset of DNA fragmentation. Degradation of NUP153 and lamin B correlated with disruption of the nuclear periphery and clustering of nuclear pores.

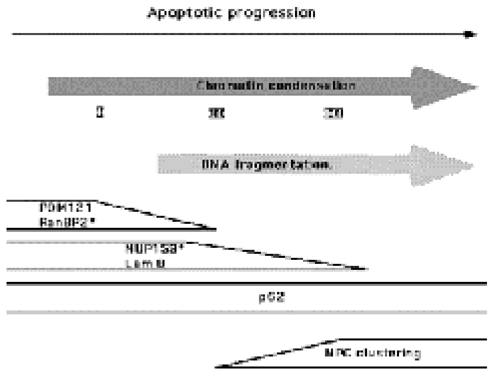

**ACKNOWLEDGEMENT**. Supported by the Swedish National Board for Laboratory Animals.

**REFERENCES.**

1. Wyllie, A.H., Kerr, J.F.R., and Currie, A.R. (1980) Int. Rev. Cytol. 68, 251-305
2. Lazebnik, Y.A., Cole, S., Cooke, C.A., Nelson, W.G., and Earnshaw, W.C. (1993) J. Cell Biol. 123(1), 7-22
